# A suite of recombinant luminescent bacterial strains for the quantification of bioavailable heavy metals and toxicity testing

**DOI:** 10.1186/1472-6750-9-41

**Published:** 2009-05-08

**Authors:** Angela Ivask, Taisia Rõlova, Anne Kahru

**Affiliations:** 1 Laboratory of Molecular Genetics, National Institute of Chemical Physics and Biophysics, Akadeemia tee 23, Tallinn, Estonia

## Abstract

**Background:**

Recombinant whole-cell sensors have already proven useful in the assessment of the bioavailability of environmental pollutants like heavy metals and organic compounds. In this work 19 recombinant bacterial strains representing various Gram-positive (*Staphylococcus aureus *and *Bacillus subtilis*) and Gram-negative (*Escherichia coli, Pseudomonas fluorescens*) bacteria were constructed to express the luminescence encoding genes *luxCDABE *(from *Photorhabdus luminescens*) as a response to bioavailable heavy metals ("lights-on" metal sensors containing metal-response elements, 13 strains) or in a constitutive manner ("lights-off" constructs, 6 strains).

**Results:**

The bioluminescence of all 13 "lights-on" metal sensor strains was expressed as a function of the sub-toxic metal concentrations enabling the quantitative determination of metals bioavailable for these strains. Five sensor strains, constructed for detecting copper and mercury, proved to be target metal specific, whereas eight other sensor strains were simultaneously induced by Cd^2+^, Hg^2+^, Zn^2+^and Pb^2+^. The lowest limits of determination of the "lights-on" sensor strains for the metals tested in this study were (μg l^-1^): 0.002 of CH_3_HgCl, 0.03 of HgCl_2_, 1.8 of CdCl_2_, 33 of Pb(NO_3_)_2_, 1626 of ZnSO_4_, 24 of CuSO_4 _and 340 of AgNO_3_. In general, the sensitivity of the "lights-on" sensor strains was mostly dependent on the metal-response element used while the selection of host bacterium played a relatively minor role. In contrast, toxicity of metals to the "lights-off" strains was only dependent on the bacterial host so that Gram-positive strains were remarkably more sensitive than Gram-negative ones.

**Conclusion:**

The constructed battery of 19 recombinant luminescent bacterial strains exhibits several novel aspects as it contains i) metal sensor strains with similar metal-response elements in different host bacteria; ii) metal sensor strains with metal-response elements in different copies and iii) a "lights-off" construct (control) for every constructed recombinant metal sensor strain. To our knowledge, no Gram-positive metal sensor expressing a full bacterial bioluminescence cassette (*luxCDABE*) has been constructed previously.

## Background

The use of microbial cells for ecotoxicological analysis as well as for the toxicological screening of various chemicals is increasing. A variety of whole-cell sensors based on natural or genetically modified microbes has been developed for this purpose. In general, there are two strategies for developing such microbial sensors: one utilizes microbes expressing the reporting signal e.g., bioluminescence constitutively ("lights-off" bacterial strains) and the other in chemical/effect-induced manner ("lights-on" sensors). The "lights-off" bacterial strains have been extensively used for toxicity testing of pure compounds as well as environmental samples [1,2] already for decades. The most popular such tests are Microtox^®^, BioTox™, LUMIStox™, ToxAlert™, which measure the effect of the sample on the luminescence of a naturally bioluminescent bacterium *Vibrio fischeri *[3].

"Lights-on" sensors are usually recombinant microbial cells containing a metal-response unit (traditionally a transcriptional regulator and its controlled promoter) fused to a promoterless reporter gene encoding for the reporting signal. Upon the presence of a bioavailable compound of interest (heavy metal or an organic compound/group of compounds), its entrance to the sensor cell and subsequent binding to the transcriptional regulator the expression of a reporter gene will take place. β-galactosidase, GFP and bioluminescence (the most favourable) have been used most often for reporter applications (reviewed in [4]). For environmental applications, LuxCDABE (a whole bacterial gene cassette required for the production of bioluminescence) is the most suitable reporter, primarily due to rapidity and ease of use. The first recombinant luminescent "lights-on" sensors were constructed in 1991 for naphthalene [5] and mercury [6]. Since then, a number of papers on bacterial strains "sensing" inorganic substances (mainly heavy metals or metalloids) but also organic compounds (e.g., benzene and its derivatives, naphthalene, polychlorinated biphenyls) have been published (reviewed in [4,7]). Currently, recombinant bacterial "lights-on" heavy metal sensors expressing bacterial bioluminescence system as a reporter include sensors for Cd, Pb [8], Hg [8-12], Cr [8], Ni, Co [13], Zn, Cu [8,14,15] and As [16]. In most of these sensors, the bioluminescence-encoding genes from *Vibrio fischeri *(that may become labile in temperatures above 30°C [4] and thus, restrict testing) has been used. Most of these papers describe just a few sensors that are often based on *Escherichia coli *[9,10,12,14] orsome other Gram-negative bacterium from the genera *Pseudomonas *or *Alcaligenes *[8,11,13,15] as a host. Thus, Gram-positive bacteria have rarely been used as hosts and no Gram-positive metal sensors expressing *luxCDABE *as a reporter have been constructed so far.

The recombinant luminescent sensor bacteria have been used for the assessment of bioavailable (the fraction entering the cells and inducing bioluminescence) metals from soils or water bodies [8,11,13,15,17]. However, due to the complexity of the environmental samples (colour, turbidity, mixed pollution) and the sensitivity of the used reporter system (bioluminescence is closely related to the energetic status of the cell [18]), several researchers [13,17,19-24] have recognized the need for relevant controls which enable accounting for unspecific effects of the sample. This paper reports on the development of a battery of recombinant bioluminescent bacterial heavy metal "lights on" sensors accompanied by corresponding bioluminescent non-specific "lights off" control strains.

## Results

### Luminescent Gram-negative strains

#### *Escherichia coli*-based strains

*Escherichia coli *is an enteric Gram-negative bacterium with the sequenced genome [25], well developed methods for genetic modification [26] and various laboratory strains available. Although the relevance of this bacterium for environmental testing is questionable, it has been widely used in the construction of various bacterial sensors [9,10,12,14,17], mainly due to its compatibility with the chemical-response elements used.

**Figure 1:**
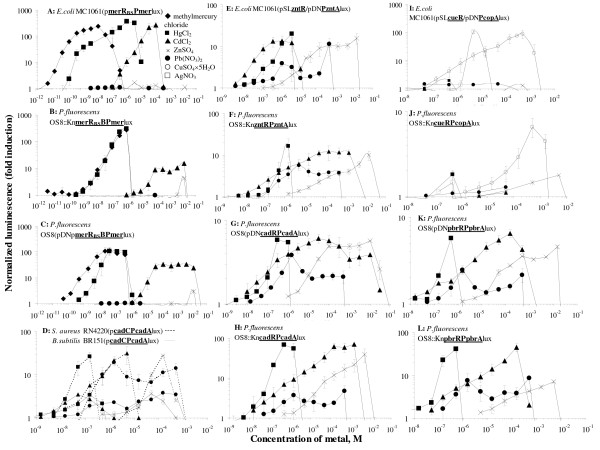
**Induction of luminescence (expressed as normalized luminescence) by heavy metals in different sensor strains**. Names of sensors and symbols for heavy metals are indicated. Data represent mean ± SD of three independent measurements.

In total three new *E. coli *MC1061 recombinant luminescent sensor strains expressing *luxCDABE *genes in response to subtoxic concentrations of bioavailable heavy metals ("lights-on" sensors) were constructed: *E. coli *MC1061(p**merR**_BS_**BPmer**lux), *E. coli *MC1061(pSL**cueR**/pDN**PcopA**lux) and *E. coli *MC1061(pSL**zntR**/pDN**PzntA**lux) (Table 1). All the strains harbour plasmid(s) containing metal-response elements from resistance systems for **Hg **(from *Serratia marcescens *plasmid [27]; **mer**), **Cu **(**cue**/**cop**) and **Zn **(**znt**) (both from *E. coli *chromosome [28,29]) fused with the *luxCDABE *cassette [see Additional file 1]. MC1061(pSL**cueR**/pDN**PcopA**lux) and MC1061(pSL**zntR**/pDN**PzntA**lux) contain two plasmids necessary for sensor function, of which one (pSL**zntR **or pSL**cueR**, respectively) is present in bacterial cells in high copy number and contains genes for the regulatory proteins (***cueR ***or ***zntR***). The other plasmid (pDNP**zntA**lux or pDN**PcopA**lux, respectively) present in medium copy number contains the *lux*-genes fused to a promoter (**P*copA ***or **P*zntA***) controlled by the regulatory proteins. Location of the genes for regulatory protein and the corresponding promoter in different plasmids yields high regulatory protein to promoter ratio necessary to reduce the unfavourably high level of leakage of the **P*copA ***and **P*zntA ***and thus, high background luminescence of those strains (Table 1). Characteristics (number of cells in the test, background luminescence, maximum induction) of the constructed sensors are presented in Table 1. Induction profiles of those sensor strains with seven tested heavy metals are presented in Figure 1 and resepective limits of determination in Table 1.

**Table 1:** Characteristics of *Escherichia coli *MC1061-based luminescent heavy metal sensors and toxicity control strains

	**Sensor strains**			**Control strains**	
	
	MC1061 (p**merR**_BS_**BPmer**lux)	MC1061 (pSL**zntR**/ pDN**PzntA**lux)	MC1061 (pSL**cueR**/ pDN**PcopA**lux)	MC1061 (pSLlux)^a^	MC1061 (pDNlux)^b^
	
**Background luminescence**^c^	230 ± 90	2940 ± 930	520 ± 70	575000 ± 35200	6500 ± 2270
**Maximum induction**^d^	400	20	100	no induction	no induction
**No of cells in the test **(100 μl)	4 × 10^7^	10^7^	8 × 10^6^	8 × 10^6^	10^7^
					
***Compound***	**Limit of determination**^e^/**Toxicity**^f^, M (mg of compound l^-1^)			**Toxicity **(2-h EC_50_^g^), M (mg of compound l^-1^)	
		
**HgCl**_2_	10^-10 ^(0.00003)/5 × 10^-6 ^(1.4)	8 × 10^-8 ^(0.02)/10^-5 ^(2.7)	no induction	10^-6 ^(0.4)	10^-6 ^(0.4)
**CH**_3_**HgCl**	8 × 10^-12 ^(0.000002)/5 × 10^-8 ^(0.01)	not tested	not tested	3 × 10^-6 ^(0.7)	not tested
**CdCl**_2_	3 × 10^-7 ^(0.06)/5 × 10^-4 ^(92)	10^-8 ^(0.003)/5 × 10^-6 ^(0.9)	no induction	4 × 10^-4 ^(70)	2 × 10^-4 ^(31)
**ZnSO**_4_^h^	no induction	5 × 10^-6 ^(0.8)/5 × 10^-2 ^(8000)	no induction	10^-2 ^(1600)	4 × 10^-3 ^(640)
**Pb(NO**_3_**)**_2_	no induction	7 × 10^-7 ^(0.2)/10^-3 ^(330)	no induction	2.4 × 10^-4 ^(79)	6 × 10^-4 ^(190)
**CuSO**_4_^i^	not tested	not tested	10^-7 ^(0.02)/10^-3 ^(240)	not tested	5.4 × 10^-4 ^(86)
**AgNO**_3_	not tested	not tested	2 × 10^-6 ^(0.3)/10^-5 ^(2.6)	not tested	2 × 10^-6 ^(0.3)

^a ^control for MC1061(p**merR**_BS_**BPmer**lux)

^b ^control for MC1061(pSL**cueR**/pDNP**copA**lux) and MC1061(pSL**zntR**/pDNP**zntA**lux)

^c ^luminescence (relative light units, RLU) of 100 μl of bacterial suspension before the test as measured by plate luminometer Fluoroskan (ThermoLabsystems). Average of at least three assays

^d ^as fold over the (non-induced) background

^e ^see equation 4 in Methods

^f ^the lowest tested concentration causing a measurable decrease in the bioluminescence of the sensor strains (read from concentration-induction curves in Figure 1)

^g ^concentration causing 50% decrease in bioluminescence production (calculated from Figure 2)

^h ^tested as ZnSO_4 _× 7H_2_O

^i ^tested as CuSO_4 _× 5H_2_O

**Figure 2:**
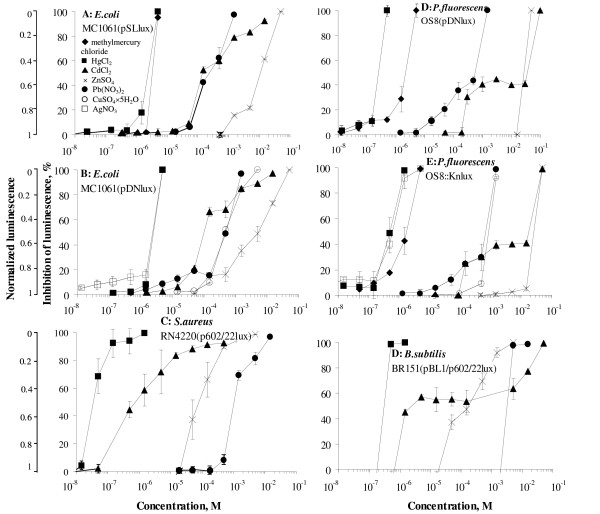
**Toxicity of heavy metals for constitutively luminescent control strains**. Toxicity of heavy metals is expressed as inhibition of bioluminescence or decrease in normalized luminescence after 2 hours incubation. Names of strains and symbols for heavy metals are indicated. Data represent mean ± SD of three independent measurements. 2-h EC_50 _values calculated from these curves are presented in Tables 1-4.

In parallel with sensors, two *E. coli *toxicity control ("lights-off") strains: MC1061(pSLlux) and MC1061(pDNlux) were constructed (Table 1). These constitutively luminescent strains lacking metal-response elements contain plasmids in which the *luxCDABE *genes were either under the *lac *promoter (pSLlux) or T7 promoter (pDNlux). Indeed, within the limits of error, the control strains were not induced by the heavy metals (Figure 2) and inhibition of bioluminescence (NL) was observed with increasing metal concentrations. Toxicity (2-h EC_50 _values) of the seven tested heavy metals to *E. coli *MC1061(pSLlux) and MC1061(pDNlux) are presented in Table 1 and respective concentration-effect curves (used for EC_50 _calculation) in Figure 2.

#### *Pseudomonas fluorescens *– based strains

*Pseudomonas fluorescens *is a non-pathogenic saphrophyte, which inhabits soil, water and plant surface, often bearing the ability to degrade various pollutants and is thus a very relevant organism for environmental analysis. *P. fluorescens *has been used as a test organism for general toxicity [30] as well as a host bacterium for constructed recombinant sensors for mercury, arsenic [19], copper [31] but also for phenolic compounds [32].

In the current study eight recombinant luminescent *P. fluorescens OS8*-based sensor strains were constructed (Tables 2 and 3). Three strains: OS8(pDN**merR**_BS_**BPmerlux**), OS8(pDN**pbrRPpbrA**lux) and OS8(pDN**cadRPcadA**lux) contained the sensor and reporter elements on a **plasmid **(about 10 copies per cell) and five sensor strains: OS8::Kn**merR**_BS_**BPmer**lux, OS8:: Kn**cueRPcopA**lux, OS8::Kn**zntRPzntA**lux, OS8::Kn**pbrRPpbrA**lux and OS8::Kn**cadRPcadA**lux contained the sensor and reporter elements as a single **chromosomal **insertion. The heavy metal response elements used originated from resistance systems for **Hg **(from *Serratia marcescens *plasmid [27]; **mer**), **Pb **(from *Ralstonia metallidurans *CH34 plasmid pMOL30 [33]; **pbr**), **Cd **(from *Pseudomonas putida *choromosome [34]; **cad**), **Cu **(**cue**/**cop**) and **Zn **(**znt**) (both from *E. coli *chromosome [28,29]) [see Additional file 1]. The sensors are characterized in Tables 2 and 3 and their induction profiles with selected heavy metals shown in Figure 1.

**Table 2:** Characteristics of *Pseudomonas fluorescens *OS8-based plasmid-containing luminescent heavy metal sensors and toxicity control strains

	**Sensor strains**			**Control strain**
	
	OS8(pDN**merR**_BS_**B** **Pmer**lux)	OS8(pDN**pbrR** **PpbrA**lux)	OS8(pDN**cadR** **PcadA**lux)	OS8(pDNlux)
	
**Background luminescence**^a^	1600 ± 540	3960 ± 1300	25900 ± 8700	64500 ± 6600
**Maximum induction**^b^	200	7	6	no induction
**No of cells in the test **(100 μl)	5 × 10^6^	4 × 10^6^	4 × 10^6^	5 × 10^6^
				
***Compound***	**Limit of determination**^c^/**Toxicity**^d^, M (mg of compound l^-1^)			**Toxicity **(2-h EC_50_^e^), M (mg of compound l^-1^)
	
**HgCl**_2_	10^-9 ^(0.0003)/10^-7 ^(0.04)	10^-7 ^(0.04)/10^-6 ^(0.4)	10^-7 ^(0.03)/10^-6 ^(0.4)	2.5 × 10^-7 ^(0.07)
**CH**_3_**HgCl**	10^-10 ^(0.00003)/10^-7 ^(0.04)	not tested	not tested	2 × 10^-6 ^(0.5)
**CdCl**_2_	5 × 10^-6 ^(0.9)/10^-2 ^(2740)	8 × 10^-7 ^(0.2)/5 × 10^-4 ^(92)	6 × 10^-8 ^(0.01)/10^-4 ^(18)	4 × 10^-2 ^(7300)
**ZnSO**_4_^f^	No induction	4 × 10^-5 ^(6.4)/5 × 10^-2 ^(8050)	8 × 10^-6 ^(1.3)/3 × 10^-2 ^(4800)	3 × 10^-2 ^(4800)
**Pb(NO**_3_**)**_2_	No induction	9 × 10^-7 ^(0.3)/10^-3 ^(500)	4 × 10^-7 ^(0.1)/10^-3 ^(500)	7 × 10^-4 ^(230)
**CuSO**_4_^g^	not tested	not tested	not tested	not tested
**AgNO**_3_	not tested	not tested	not tested	not tested

^a ^luminescence (relative light units, RLU) of 100 μl of bacterial suspension before the test as measured by plate luminometer Fluoroskan (ThermoLabsystems). Average of at least three assays

^b ^as fold over the (non-induced) background

^c ^see equation 4 in Methods

^d ^the lowest tested concentration causing a measurable decrease in the bioluminescence of the sensor strains (read from concentration-induction curves in Figure 1)

^e ^concentration causing 50% decrease in the bioluminescence production (calculated from Figure 2)

^f ^tested as ZnSO_4 _× 7H_2_O

^g ^tested as CuSO_4 _× 5H_2_O

**Table 3:** Characteristics of *Pseudomonas fluorescens *OS8-based chromosomal luminescent heavy metal sensors and toxicity control strains

	**Sensor strains**					**Control strain**
	
	OS8::Kn**merR**_BS_**BPmer**lux	OS8::Kn**cueRPcopA**lux	OS8::Kn**zntRPzntA**lux	OS8::Kn**pbrR** **PpbrA**lux	OS8::Kn**cadR** **PcadA**lux	OS8::Knlux
	
**Background luminescence**^a^	1290 ± 240	1250 ± 180	3530 ± 1570	84 ± 35	2860 ± 320	600–1400^b^
**Maximum induction**^c^	400	7	20	50	80	no induction
**No of cells in the test **(100 μl)	2 × 10^7^	3 × 10^7^	6 × 10^6^	3 × 10^6^	3 × 10^7^	3 × 10^6 ^– 3 × 10^7b^
						
***Compound***	**Limit of determination**^d^/**Toxicity**^e^, M (mg of compound l^-1^)					**Toxicity **(2-h EC_50_^f^), M (mg compound l^-1^)
	
**HgCl**_2_	3 × 10^-9 ^(0.0008)/5 × 10^-6 ^(1.3)	no induction	3 × 10^-7 ^(0.08)/5 × 10^-6 ^(1.4)	3 × 10^-8 ^(0.008)/10^-6 ^(0.4)	2 × 10^-8 ^(0.005)/10^-6 ^(0.4)	5 × 10^-7 ^(0.14)
**CH**_3_**HgCl**	3 × 10^-9 ^(0.0008)/5 × 10^-6 ^(1.3)	not tested	not tested	not tested	not tested	2 × 10^-6 ^(0.58)
**CdCl**_2_	5 × 10^-5 ^(9.2)/5 × 10^-2 ^(9150)	no induction	8 × 10^-8 ^(0.02)/10^-2 ^(2750)	8 × 10^-8 ^(0.02)/5 × 10^-4 ^(92)	3 × 10^-8 ^(0.006)/5 × 10^-3 ^(915)	2 × 10^-2 ^(3660)
**ZnSO**_4_^g^	no induction	no induction	3 × 10^-5 ^(4.8)/5 × 10^-2 ^(8050)	2 × 10^-5 ^(3.2)/10^-2 ^(2420)	4 × 10^-6 ^(0.6)/2 × 10^-2 ^(3220)	3.5 × 10^-2 ^(5640)
**Pb(NO**_3_**)**_2_	no induction	no induction	4 × 10^-7 ^(0.1)/10^-3 ^(500)	2 × 10^-7 ^(0.07)/10^-3 ^(500)	3 × 10^-7 ^(0.1)/10^-3 ^(500)	7 × 10^-4 ^(231)
**CuSO**_4_^h^	not tested	5 × 10^-5 ^(8)/10^-3 ^(240)	not tested	not tested	not tested	8 × 10^-3 ^(1280)
**AgNO**_3_	not tested	no induction	not tested	not tested	not tested	5 × 10^-7 ^(0.083)

^a ^luminescence (relative light units, RLU) of 100 μl of bacterial suspension before the test as measured by plate luminometer Fluoroskan (ThermoLabsystems). Average of at least three assays

^b ^cells at different optical densities (see Methods) yielded different RLU values and number of viable cells

^c ^as fold over the (non-induced) background

^d ^see equation 4 in Methods

^e ^the lowest tested concentration causing a measurable decrease in the bioluminescence of the sensor strains (read from concentration-induction curves in Figure 1)

^f ^concentration causing 50% decrease in the bioluminescence production (calculated from Figure 2)

^g ^tested as ZnSO_4 _× 7H_2_O

^h ^tested as CuSO_4 _× 5H_2_O

In addition to sensors, two toxicity control ("lights-off") strains were constructed (Tables 2 and 3). One of these, *P. fluorescens *OS8(pDNlux), contained the *luxCDABE *cassette under the T7 promoter on a plasmid and the other, *P. fluorescens *OS8::lux, contained the *luxCDABE *under the *lac *promoter as a chromosomal insertion. In both strains the bioluminescence was constitutive as within the limits of error they were not induced by the heavy metals (Figure 2). Toxicity (2-h EC_50 _values) of the tested metals to *P. fluorescens *OS8(pDNlux) and OS8::lux are presented in Tables 2 and 3 and respective concentration-effect curves in Figure 2.

### Luminescent Gram-positive strains

*Bacillus subtilis *and *Staphylococcus aureus *were used to construct Gram-positive heavy metal sensor strains. *Bacillus subtilis *is a spore-forming bacterium common in soils and thus a relevant environmental test organism. Recombinant *B. subtilis *has earlier been used as a model Gram-positive bacterium for toxicity testing [35] as well as a host for a recombinant bacterial cadmium sensor [36]. *Staphylococcus aureus*, although not an environmentally relevant bacterium, it may be a medically important model organism as it is an opportunistic human pathogen often bearing multiple antibiotic resistance.

Two Gram-positive sensor strains, *S. aureus *RN4220(p**cadCPcadA**lux) and *B. subtilis *BR151(p**cadCPcadA**lux), in which the *luxCDABE *genes were under the control of heavy metal-inducible ***cadC***-**P*cadA ***system from *S. aureus *plasmid pI258 [36] were constructed. The *luxCDABE *genes used were modified from *Photorhabdus luminescens *by optimizing the codon usage and ribosome binding sites for Gram-positive bacteria [37]. The constructed sensors are characterized in Table 4 and their induction profiles with Hg^2+^, Cd^2+^, Pb^2+^, Zn^2+ ^are presented in Figure 1.

**Table 4:** Characteristics of *Staphylococcus aureus *and *Bacillus subtilis*-based luminescent heavy metal sensors and toxicity control strains

	**Sensor strains**		**Control strains**	
	
	*S. aureus *RN4220 (p**cadCPcadA**lux)	*B. subtilis *BR151 (p**cadCPcadA**lux)	*S. aureus *RN4220 (p602/22lux)^a^	*B. subtilis *BR151 (pBL1/p602/22lux)^b^
	
**Background luminescence**^c^	81 ± 30	84 ± 30	750 ± 70	120 ± 40
**Maximum induction**^d^	30	4	no induction	no induction
**No of cells in the test **(100 μl)	10^7^	10^7^	10^7^	10^7^
				
***Compound***	**Limit of determination**^e^/**Toxicity**^f^, M (mg of compound l^-1^)		**Toxicity **(2-h EC_50_^g^), M (mg of compound l^-1^)	
	
**HgCl**_2_	10^-8 ^(0.003)/5 × 10^-7 ^(0.1)	4 × 10^-8 ^(0.01)/5 × 10^-7 ^(0.1)	5 × 10^-8 ^(0.01)	10^-7 ^(0.04)
**CdCl**_2_	4 × 10^-8 ^(0.007)/10^-5 ^(2.75)	10^-8 ^(0.002)/10^-7 ^(0.027)	8 × 10^-7 ^(0.2)	3 × 10^-6 ^(0.6)
**ZnSO**_4_^h^	2 × 10^-5 ^(3.2)/5 × 10^-4 ^(81)	10^-5 ^(1.6)/10^-4 ^(24)	8 × 10^-5 ^(13)	10^-4 ^(22)
**Pb(NO**_3_**)**_2_	10^-7 ^(0.03)/10^-3 ^(500)	10^-7 ^(0.03)/10^-3 ^(500)	10^-3 ^(530)	3 × 10^-3 ^(990)

^a ^control for RN4220(p**cadCPcadA**lux)

^b ^control for BR51(p**cadCPcadA**lux)

^c ^luminescence (relative light units, RLU) of 100 μl of bacterial suspension before the test as measured by plate luminometer Fluoroskan (ThermoLabsystems). Average of at least three assays.

^d ^as fold over the (non-induced) background

^e ^see equation 4 in Methods

^f ^the lowest tested concentration causing a measurable decrease in the bioluminescence of the sensor strains (read from concentration-induction curves in Figure 1)

^g ^concentration causing 50% decrease in the bioluminescence production (calculated from Figure 2)

^h ^tested as ZnSO_4 _× 7H_2_O

Two constitutively luminescent Gram-positive control ("lights-off") strains *S. aureus *RN4220(p602/22lux) and *B. subtilis *BR151(pBL1/p602/22lux) in which the optimized *luxCDABE *[37] is under the *lac *promoter, were constructed (Table 4). However, analogously to earlier studies [38] the stability of the plasmids with genes encoding for bioluminescence was relatively poor in Gram-positive bacterial strains as frequent loss of plasmids especially in strains with higher background luminescence, was observed. Even, no transformants were obtained when plasmid p602/22lux was transformed into *B. subtilis *BR151. This problem was solved and the relative stability of the bioluminescence encoding genes was achieved when the plasmid was transformed to *B. subtilis *strain containing a helper plasmid pBL1 (expresses LacIrepressor) [38]. Toxicity (2-h EC_50 _values) of Hg^2+^, Cd^2+^, Pb^2+ ^and Zn^2+ ^to *S. aureus *RN4220(p602/22lux) and *B. subtilis *BR151(pBL1/p602/22lux) are presented in Table 4 and respective concentration-effect curves in Figure 2.

## Discussion

We constructed a set of Gram-negative and Gram-positive bioluminescent bacterial sensors in which the *luxCDABE *genes are regulated either by metal-response elements ("lights-on" metal sensors) or expressed in a constitutive manner ("lights-off" control strains). The developed set of bacterial sensors is original in several aspects:

• both Gram-negative (*E. coli*, *P. fluorescens*) and Gram-positive (*S. aureus*, *B. subtilis*) bacteria were used as hosts for heavy metal sensors and *luxCDABE *genes from *Photorhabdus luminescens *(thermostability > 30°C) were used as a reporter system;

• this is the first paper on construction and characterization of Gram-positive *lux*-cassette expressing sensors for Cd, Hg, Zn and Pb;

• when possible, similar metal-response elements were used in different bacterial hosts, to evaluate the effect of the host bacterium on the specificity and sensitivity of the constructed sensor;

• similar metal-response elements were expressed both in plasmid and in chromosome of *P. fluorescens*, to determine the effect of the copy number of the *lux *cassette-fused metal-response elements on the performance of the sensors;

• a constitutively luminescent control ("lights-off" construct) was constructed for each heavy metal sensor and used to account for unspecific effects of the samples on bacterial bioluminescence. These control bacteria can also be used for general toxicity testing.

### Properties of "lights-off" control strains

The constitutively luminescent "lights-off" control strains had always higher background bioluminescence than the respective control strains (Tables 1, 2, 3, 4). The highest background luminescence (600 000 RLU) was measured for *E. coli *MC1061(pSLlux) probably due to the location of the *luxCDABE *on a high-copy-number plasmid. As the rationale of including the control strains in the assay is to take into account the possible non-specific effects (e.g., toxicity) of the sample on bacterial bioluminescence, the high initial bioluminescence allowing more accurate measurement of the inhibition of bioluminescence, is certainly a bonus. By decreasing the plasmid copy number, the background bioluminescence of the "lights-off" strains clearly decreased. The bioluminescence of *E. coli *MC1061(pDNlux) containing a medium-copy-number plasmid was about 100-fold lower than that of *E. coli *MC1061(pSLlux) (Table 1). The same trend was observed for *P. fluorescens *as the background luminescence of OS8(pDNlux) (*luxCDABE *in about 10 copies) was about 100-fold higher than that of the OS::Knlux (a single copy of *luxCDABE*) (Tables 2 and 3). However, the background luminescence value did not affect the sensitivity of the "lights-off" strains to heavy metals: the EC_50 _values of differently luminescent *E. coli *and *P. fluorescens *control strains were similar (Tables 1, 2 and 3). Almost no effect of bioluminescence on bacterial sensitivity to heavy metals has also been demonstrated earlier by showing that a *lux*-marked *Burkholderia sp. *had similar sensitivity to Zn and Cu as the wild (not luminescent) strain in dehydrogenase assay [39]. In addition, we showed that the toxicity of Hg, methylmercury, Pb and Ag to the both Gram-negative bacteria (*E. coli *and *P. fluorescens*) was similar. However, *E. coli *proved more sensitive than *P. fluorescens *towards Zn (about 3-fold), Cu (about 15-fold) and Cd (about 100-fold) (Tables 1, 2 and 3). Indeed, relatively low sensitivity of a *Pseudomonas putida *towards Cd (about 3 orders of magnitude lower than the sensitivity of *E. coli*) in a growth ihibition assay has been shown earlier [40].

Comparison of Gram-negative and -positive control strains showed that the luminescence of the latter was always lower (Tables 1, 2, 3, 4). This could be either due to the poor expression of bioluminescence-encoding genes in these strains or partial loss of *lux*-containing plasmid in the bacterial cultures. In addition, Gram-positive strains were remarkably more sensitive to the tested heavy metals than Gram-negative (EC_50 _values in Tables 1, 2, 3, 4). Interestingly, other authors [41] have shown that the toxiciy of Hg was similar to *E. coli *and *B. subtilis *whereas we showed that the latter was at least 20-fold more senistive than the former.

### Properties of heavy metal sensor strains: background luminescence

Differently from "lights-off" constructs, in metal sensor strains the lower background luminescence is an advantage as it ensures a more pronounced induction at lower metal concentrations (in the case of a strain with high background luminescence this high value must be exceeded at least twice to be equivalent to LOD). Theoretically, the sensor strains should be bioluminescent (induced) only in the presence of intracellular heavy metals, which bind on the regulatory protein. However, background luminescence values in the sensor strains were also registered in the absence of added heavy metals (Tables 1, 2, 3, 4). The reason for this could be either the leakage of the promoter controlling the expression of *lux*-genes and/or as proposed by [42] metal impurities of the test medium. Indeed, the optimized medium used in our study (see Methods), contained trace amounts of Cd (0.0001 mg l^-1^), Zn (0.035 mg l^-1^), Cu (0.0025 mg l^-1^) and Pb (0.003 mg l^-1^). However, these concentrations were more than 5-460-fold lower than the LODs of the sensors, depending on the strain (Tables 1, 2, 3, 4). Also, the background luminescence of the sensor strains (varying among the strains; Tables 1, 2, 3, 4) did not correlate with their sensitivities to the metals present as impurities in the test medium. Thus, the most probable reason for the background luminescence in the sensor strains is the leakage of the promoter controlling the expression of *luxCDABE *genes. Indeed, the *E. coli *MC1061(p**merR**_BS_**BPmer**lux) had remarkably lower background than the other *E. coli *strains and it is well known that the regulatory protein in this sensor, **MerR**, is a strong repressor in the absence of mercury [43,44]. At the same time, the regulatory protein **ZntR **in MC1061(pSL**zntR**/pDN**PzntA**lux) strain with high background luminescence, has not been clearly shown to act as a repressor in the absence of zinc [29]. In addition to the nature of the metal-response elements, also the number of copies in which the *lux-*cassette and linked metal-response elements were present in the cells affected the background luminescence (*P. fluorescens *plasmid-bearing *versus *chromosomal strains, Tables 2 and 3). As a rule, the higher the background luminescence the lower the maximum induction of the sensors (Tables 1, 2, 3, 4). This could be due to the depletion of energy in the bacterial cells at high bioluminescence values as the emission of bioluminescence requires high amounts of cellular energy [45]. However, the background luminescence of the metal sensor strains did not affect their metal sensitivity (LOD) (Tables 1, 2, 3, 4).

### Properties of heavy metal sensor strains: sensitivity and specificity

The sensor strains were characterized for their sensitivity and specificity using selected environmentally relevant heavy metals. The induction of luminescence was measured after 2 hours incubation that was selected according to our optimization studies (T.Rõlova, personal communication) and similar studies by other authors [17]. Interestingly, only one of the sensors, *P. fluorescens *OS8::Kn**cueRPcopA**lux was single metal (Cu) specific (Figure 1J). The *E. coli *MC1061(pSL**cueR**/pDN**PcopA**lux) expressing similar metal-response elements was in addition to Cu induced also by Ag, but in relatively high (environmentally irrelevant) concentrations (Figure 1I). The sensors expressing Hg-response elements (*E. coli *MC1061(p**merR**_BS_**BPmer**lux), *P. fluorescens *OS8(pDN**merR**_BS_**BPmer**lux) and OS8::Kn**merR**_BS_**BPmer**lux)) showed also relatively high specificity, by being induced only with inorganic and organic mercury compounds and Cd. However, induction with the latter occurred at concentrations 3-4 orders of magnitude higher than in the case of mercury and yielded remarkably lower inductions (Figure 1A–C). These data are in accordance with [46-48]. It is interesing to note that *E. coli *MC1061(p**merR**_BS_**BPmer**lux) and *P. fluorescens *OS8(pDN**merR**_BS_**BPmer**lux) were detecting methylmercury at about one order of magnitude lower concentrations than HgCl_2 _(Tables 1 and 2). Similar results for the same metal-response elements have been shown in [49] and is most probably caused by higher bioavailability of the organic mercury salt. On the other hand, both inorganic mercury and methylmercury were of similar toxicity to *E. coli *MC1061(pSLlux) or the latter was even less toxic to *P. fluorescens *control strains (Figure 2A), indicating that the elevated sensitivity towards methylated mercury occurs only at sub-toxic concentrations.

Sensor bacteria carrying the metal-response elements from Zn-resistance system of *E. coli *(*E. coli *MC1061(pSL**zntR**/pDN**PzntA**lux) and *P. fluorescens *OS8::Kn**zntRPzntA**lux), Cd-resistance system from *Pseudomonas putida *(*P. fluorescens *OS8(pDN**cadRPcadA**lux) and OS8::Kn**cadRPcadA**lux), Pb-resistance system from *Ralstonia metallidurans *(*P. fluorescens *OS8(pDN**pbrRPpbrA**lux), OS8::Kn**pbrRPpbrA**lux) or Cd-resistance system from *S. aureus (S. aureus *RN4220(p**cadCPcadA**lux) and *B. subtilis *BR151(p**cadCPcadA**lux)) responded to four of the selected seven heavy metal salts: HgCl_2_, CdCl_2_, Pb(NO_3_)_2 _and ZnSO_4 _(Figure 1, Tables 1, 2, 3, 4) and the order of induction in all the cases was Hg^2+ ^≈ Cd^2+ ^< Pb^2+ ^< Zn^2+^. These unspecific responses were somewhat expected as earlier studies have shown similar results e.g. for Zn-resistance determinant from *E. coli *chromosome [14,50] and to our knowledge, no specific Cd-response system has been found. Unspecificity of several metal-response systems could be due to the evolution of these metal resistance systems to be induced by several heavy metals in order to detoxify them, or simply because the metal-binding proteins in these systems are unable to differentiate between different bi-valent metal ions [51].

Comparison of sensitivities of the current metal sensor strains with previously constructed ones (see below) showed that some of the current sensors were detecting very low levels of metals (lowest LOD values reported so far). However, variability of test results may occur between different papers due to different experimental conditions, which may affect the speciation and thus, bioavailability of metals. Nevertheless, our comparison showed that the strain *E. coli *MC1061(pSL**cueR**/pDN**PcopA**lux) detected 20-fold lower concentrations of Cu than similar previously published bacterial strains [11] and two strains – *E. coli *MC1061(p**merR**_BS_**BPmer**lux) and *B. subtilis *BR151(p**cadCPcadA**lux) – were similar in sensitivity with the most sensitive previously reported strains for Hg and Cd, respectively [10,11,16,52].

### Comparison of sensor constructs in different host bacteria

Performance of different types of recombinant bacteria carrying similar metal-response elements was compared. Comparison of the two Gram-negative host bacteria *E. coli *and *P. fluorescens *showed that the *E. coli*-based metal sensors were somewhat more sensitive. For example, *E. coli *MC1061(pSL**cueR**/pDN**PcopA**lux) was about 300-fold more sensitive to Cu than *P. fluorescens *OS8::Kn**cueRPcopA**lux and *E. coli *MC1061(p**merR**_BS_**BPmer**lux) was induced by at least 10-fold lower concentrations of Hg^2+^, methylmercury and Cd^2+ ^than *P. fluorescens *OS8(pDN**merR**_BS_**BPmer**lux). This and the lower toxicity of some heavy metals to *P. fluorescens *compared with *E. coli *could be explained by different cellular metal homeostasis (e.g., the presence and nature of metal transporters) of the host bacteria. Indeed, the effect of metal transporters on the sensitivity of bacterial heavy metal sensors has been shown [53]. On the other hand, there were no remarkable differences in LOD values of the two Gram-positive sensor strains *S. aureus *RN4220(p**cadCPcadA**lux) and *B. subtilis *BR151(p**cadCPcadA**lux) carrying similar metal-response elements (Table 4). Thus, differently from "lights-off" strains, the sensitivity of the "lights-on" sensors depends on the nature of the metal sensing element rather than on the bacterial host. Indeed, the strains with metal-response elements from Hg resistance system were the most sensitive to Hg and the least sensitive to Cd regardless of the host strain.

Different location of the metal-response elements in *P. fluorescens *(on a plasmid present in the cells in about 10 copies or as a single chosomosmal insertion) was chosen to clarify whether lowering the copy number of the metal-response elements and *lux *genes will yield higher sensitivity, as previously shown for stress-induced bacterial strains [54]. However, our data on *P. fluorescens *with chromosomal insertions showed lower background luminescence but not higher sensitivity than the respective plasmid-containing strains (Figure 1B, C).

### Possible applications

As shown above, all the constructed metal sensor strains showed concentration-dependent linear increase in bioluminescence at sub-toxic concentrations of heavy metals. Moreover, the increased bioluminescence of the sensor implies that the toxic metal has crossed the biological membrane and thus, has a potential to accumulate in the food chain. In addition, the limits of determination of most of the constructed sensors under our test conditions were low enough to detect even the EC permitted limit values for Hg, Cd, Cu and Pb in drinking water (1, 5, 5000 and 10 μg l^-1^, respectively; Directive 98/83 EC).

Currently the most challenging field of biosensor applications is the evaluation of bioavailable amounts of heavy metals in complex environmental matrices [55,56] for risk assessment purposes. However, the use of microbial sensors for environmental analysis is currently restricted mostly due to the lack of specificity. This drawback may be overcome by the combined use of several strains with different specificities and sensitivities. In addition, Cu and Zn-sensors have already proven very useful in other fields, e.g. in quantification of solubilized bioavailable metals from metal-containing nanoparticles [57-59].

## Conclusion

As a result of this work, a battery of 19 recombinant luminescent bacterial strains was constructed which exhibits several novel aspects as it contains i) metal sensor strains with similar metal-response elements in different host bacteria; ii) metal sensor strains with metal-response elements in different copies and iii) a "lights-off" construct (control) for every constructed recombinant metal sensor strain. Thus, this set of sensors may be applicable for testing both, bioavailability of heavy metals and general toxicity.

## Methods

### Construction of plasmids and strains

Bacterial strains and plasmids used in this study are listed and their detailed characterization is provided in Additional file [see Additional file 1]. All the constructed sensors are based on components of heavy metal resistance systems (metal-response elements) and *luxCDABE *genes from *Photorhabdus luminescens *[60] (reporter).

### Gram-negative strains

#### *Escherichia coli*-based sensors and the respective control strains

Four new plasmids were constructed and electroporated [26] into *E. coli *MC1061 [61]. The obtained plasmid constructs were verified by multiple restriction analysis.

Plasmid p**merR**_BS_**BPmer**lux was constructed by ligating the 4600 bp BamHI-NheI-digested and Klenow-treated fragment of pmerR_BS_Bluc [49] and 3200 bp EcoRI-XcmI-digested and T4-treated fragment of pTetlux [62].

Plasmid pDNP**copA**lux was constructed by replacing the *lucFF *gene in pSLcueRPcopAluc [63] by *luxCDABE *genes. For that, 3900 bp BamHI-NheI fragment of pSLcueRPcopAluc was ligated with a BamHI-NheI-digested PCR fragment of *luxCDABE *(primers: 5' TTAAGGATCCGCAAATATGACTAAAAAAATT 3' and 5' ATATGCTAGCACTATCAAACGCTTCGGTTAA 3'; pmerR_BS_BPmerlux was used as a template). The resulting plasmid pSLcueRPcopAlux was digested with NaeI and the fragment containing the promoter of *copA *and the *luxCDABE *was inserted into BamHI-digested and Klenow-treated pDN18N [64]. The resulting plasmid pDNPcopAlux was electroporated into *E. coli *MC1061(pSLcueR) [63].

Plasmid pSL**zntR **was constructed by self-ligating the 3500 bp NgoMI fragment of pSLzntluc [50] containing the *zntR *gene. To construct pDN**PzntA**lux, first an intermediate pSLzntRPzntAlux was constructed by ligating the BamHI-NheI-digested pSLzntluc (containing the promoter of *zntA*) and BamHI-NheI-digested PCR fragment of *luxCDABE *(see above). A NaeI fragment of pSLzntRPzntAlux containing the promoter of *zntA *and *luxCDABE *genes was then ligated with BamHI-digested and Klenow-treated pDN18N [64] resulting in pDNPzntAlux. The plasmids pSLzntR and pDNPzntAlux were tandemly electroporated into *E. coli *MC1061 [61].

In addition to those four newly constructed plasmids, the previously constructed plasmids pSLlux and pDNlux [32] were electroporated into *E. coli *MC1061 to obtain control strains MC1061(pSLlux) and MC1061(pDNlux).

#### *Pseudomonas fluorescens*-based sensor and the respective control strains

Both, chomosomal insertion- and plasmid-containing strains of *P. fluorescens *OS8 were constructed.

### Plasmid-containing strains

Altogether three new broad-host range sensor plasmids were constructed and electroporated into *P. fluorescens *OS8 [65] by standard methods [26]. The plasmid constructs were verified by multiple restriction analysis.

Plasmid pDN**merR**_BS_**BPmer**lux was constructed by digesting the pmerR_BS_BPmerlux with PvuII and ligating with BamHI-digested and Klenow-treated pDN18N [64].

Plasmid pDN**pbrRPpbrA**lux was constructed by ligating the BamHI-XhoI-digested pSLlux [32] and BamHI-XhoI-digested 650 bp PCR product containing *pbrR *and promoter of *pbrA *(primers: 5' ATATCTCGAGGGGAGCCTTACCGGCAGAACAGCGA 3'and 5' TTAAGGATCCCCTCATGGCAACCCCTTGTGTGTATTCA 3'; *Ralstonia metallidurans *CH34 cell extract prepared as in [50] was used as a template). The resulting plasmid pSLpbrRPpbrAlux was digested with PvuII-NaeI and ligated with BamHI-digested and Klenow-treated pDN18N [64] to obtain pDNpbrRPpbrAlux.

Plasmid pDN**cadRPcadA**lux was constructed by ligating the BamHI-BspEI-digested pSLlux [32] and BamHI-AgeI-digested 550 bp PCR fragment containing *cadR *and promoter of *cadA *(primers 5' ATATACCGGTAAGCTTTTGGCTTAATGCCCGTG 3' and 5' TTAAGGATCCCATGGGGTCATCCTTAATTTGAGCC 3' were used; cell extract of *Pseudomonas putida *PaW85 prepared as in [50] was used as a template). The resulting plasmid pSLcadRPcadAlux was digested with XhoI-SalI, Klenow-treated and ligated with BamHI-digested and Klenow-treated pDN18N [64] to obtain pDNcadRPcadAlux.

The control strain OS8(pDNlux) was from [32].

### Strains with chromosomal insertions

Chromosomal insertions were done by Tn*5 *mini-transposonmutagenesis method [66]. Transposon-carrying suicide plasmids were constructed (see below) and transferred into the recipient strains by conjugation.

### Construction of suicide plasmids

All suicide plasmids were constructed in the *E. coli *strain SY372 λpir [67] and were based on pTCR241 [68]. The pTCR241 was modified as follows: 1500 and 2500 bp NotI fragments between terminal ends of the Tn5 were removed and replaced by kanamycin resistance gene from pET26b(+) (Novagen) and the resulting plasmid was desginated pTCRKn.

For the construction of pTCRKnpbrRPpbrAlux, pTCRKnmerR_BS_BPmerlux and pTCRKnzntRPzntAlux, 6900, 8200 and 7000 bp PvuII fragments of pSLpbrRPpbrAlux, pmerR_BS_BPmerlux and pSLzntRPzntAlux and for the construction of pTCRKncadRPcadAlux and pTCRKncueRPcopAlux, 6750 and 6500 bp SalI-XhoI-digested and Klenow-treated fragments of pSLcadRPcadAlux and pSLcueRPcopAlux were inserted into StuI-site of pTCRKn situated between the terminal ends of the Tn5 before the kanamycin resistance encoding gene. All the fragments inserted into pTCRKn contained respective metal-response elements and *luxCDABE *genes. For the construction of pTCRKnlux, an intermediate plasmid was constructed by inserting SalI-NruI fragment of pSLlux (*luxCDABE *genes under the *lac *promoter) into SalI-StuI-digested pSL1190. From the resulting plasmid a PvuII fragment (*lac *promoter and *luxCDABE*) was inserted into StuI-digested pTCRKn. The plasmid constructs were verified by multiple restriction analysis

### Chromosomal insertions to *P. fluorescens *OS8

For chromosomal insertions, *E. coli *S17-1 λ pir [69] was transformed with the appropriate suicide plasmid by CaCl_2 _method [26] to obtain the donor strains. The donor strain was grown overnight in LB medium (per liter: 10 g of tryptone, 10 g of NaCl, 5 g of yeast extract [26]) with 100 μg ml^-1 ^of ampicillin and mixed with the overnight culture (in LB) of the recipient *P. fluorescens *OS8 at a ratio 1:2 (20 μl: 40 μl). 5 ml of 10 mM MgSO_4 _was added to the mixture and the cells were collected on a 0.45 μm membrane filter. The filter was incubated on LB agar plate for 8–18 h to allow conjugation after which the cells on the filter were resuspended in 5 ml of 10 mM MgSO_4_. 100–500 μl of the resulting mixture was plated onto selective M9 minimal medium (per liter: 6 g of Na_2_HPO_4_, 3 g of KH_2_PO_4_, 0.5 g of NaCl, 1 g of NH_4_Cl) agar (1.5%) plates supplemented with 4 g l^-1 ^of glucose and microelements (final concentration per liter: 26.9 mg of MgO, 5 mg of CaCO_3_, 0.13 ml of conc. HCl, 11.3 mg of FeSO_4 _× 7H_2_O, 3.6 mg of ZnSO_4 _× 7H_2_O, 2.8 mg of MnSO_4 _× 4H_2_O, 0.65 mg of CuSO_4 _× 5H_2_O, 0.7 mg of CoSO_4 _× 7H_2_O, 0.15 mg of H_3_BO_3_; the mixture was prepared as 400-fold concentrate). Selectivity of the plates was obtained by supplementing the medium with 100 μg ml^-1 ^of kanamycin. Strains with chromosomal insertions of metal-response elements and *luxCDABE *genes from pTCRKnpbrRPpbrAlux, pTCRKnmerR_BS_BPmerlux, pTCRKnzntRPzntAlux, pTCRKncadRPcadAlux and pTCRKncueRPcopAlux were designated as OS8::Kn**pbrRPpbrA**lux, OS8::Kn**merR**_BS_**BPmer**lux, OS8::Kn**zntRPzntA**lux, OS8::Kn**cadRPcadA**lux and OS8::Kn**cueRPcopA**lux, respectively. The strain with chromosomal insertion of *luxCDABE *from pTCRKnlux was designated as OS8::Knlux.

### Gram-positive strains

Plasmids p**cadCPcadA**lux and p602/22lux were constructed by inserting the SmaI-SpeI-digested 5700 bp *luxCDABE *from pSB2025 (translational signals before the genes have been optimized for Gram-positive bacteria) [37,70] into NheI-BamHI-digested (BamHI-digested end was filled with Klenow fragment) pTOO24 [36] or p602/22 [71], respectively. The plasmid constructs were verified by multiple restriction analysis

For the construction of Gram-positive metal sensor and control strains, the plasmids p**cadCPcadA**lux and p602/22lux were inserted into *Staphylococcus aureus *RN4220 [72] and *Bacillus subtilis *BR151 [73] or BR151(pBL1) [38] by electroporation [74,75].

### Cultivation of bacteria

The constructed strains were stored as frozen glycerol (15%) stocks at -70°C. Prior to testing, bacteria were grown on LB [26] agar plates containing appropriate antibiotics (see below). For the test, bacteria were cultivated in M9 medium (per liter: 6 g of Na_2_HPO_4_, 3 g of KH_2_PO_4_, 0.5 g of NaCl, 1 g of NH_4_Cl, 0.25 g of MgSO_4 _× 7H_2_O, 0.01 g of CaCl_2_; [26]) supplemented with glucose (final concentration 1 g l^-1^) and acid hydrolysate of casein (cas-AA; LabM; final concentration 5 g l^-1^), HMM medium (per liter: 8.4 g of MOPS at pH 7.2, 3.7 g of KCl, 0.53 g of NH_4_Cl, 0.07 g of MgSO_4 _× 7H_2_O, 0.21 g of glycerol-2-phosphate, 0.0003 g of FeCl_3 _× 6H_2_O; all the components were prepared as 10 or 100-fold concentrated stocks, filter-sterilized and stored at +4°C except for glycerol-2-phosphate, which was stored at -20°C; [76]) supplemented with glucose (final concentration 1 g l^-1^) and cas-AA (final concentration 5 g l^-1^) or LB medium. The culture media were supplemented with antibiotics as follows: ampicillin 100 μg ml^-1 ^(*E. coli *MC1061(p**merR**_BS_**BPmer**lux), MC1061(pSLlux)), ampicillin 100 μg ml^-1 ^plus tetracycline 10 μg ml^-1 ^(*E. coli *MC106 (pSL**cueR**/pDN**PcopA**lux), MC1061(pSL**zntR**/pDN**PzntA**lux)), tetracycline 10 μg ml^-1 ^(MC1061(pDNlux)) and 20 μg ml^-1 ^(*P. fluorescens *OS8(pDN**merR**_BS_**BPmer**lux), OS8(pDN**pbrRPpbrA**lux), OS8(pDN**cadRPcadA**lux), OS8(pDNlux)), kanamycin 50 μg ml^-1 ^(*S. aureus *RN4220(p**cadCPcadA**lux), RN4220(p602/22lux) and *B. subtilis *BR151(p**cadCPcadA**lux)) or 100 μg ml^-1 ^(*P. fluorescens *OS8::Kn**merR**_BS_**BPmer**lux, OS8::Kn**cueRPcopA**lux, OS8::Kn**zntRPzntA**lux, OS8::Kn**pbrRPpbrA**lux, OS8::Kn**cadRPcadA**lux, OS8::Knlux), kanamycin 50 μg ml^-1 ^plus erythromycin 10 μg ml^-1 ^(BR151(pBL1/p602/22lux)). All cultivations were performed on a shaker at 200 rpm, 30°C.

M9 medium with the above-mentioned supplements was used to cultivate *E. coli *and *P. fluorescens *strains when they were use to test Cd, Hg, methylmercury, Zn, Cu or Ag. HMM medium with supplements was used to cultivate *E. coli *and *P. fluorescens *if they were used to test Pb. The cultures in exponential phase (OD_600 _= 0.6–0.8) were diluted with the respective test media until OD_600_~0.1 except the four *P. fluorescens *strains with chromosomal insertions, which were cultivated until the OD_600 _of 0.8 (OS8::Kn**cadRPcadA**lux and OS8::Kn**cueRPcopA**lux), 0.4 (OS8::Kn**merR**_BS_**BPmer**lux) and 0.2 (OS8::Kn**zntRPzntA**lux).

LB medium was used to cultivate *S. aureus *and *B. subtilis *strains. The cultures in exponential phase (OD_600 _= 0.6–1) were then washed twice with either glucose and cas-AA supplemented M9 medium (further test with Hg, Zn or Cd) or cas-AA and glucose-supplemented HMM (further test with Pb) and diluted until OD_600_~0.1. The number of bacterial cells in the test was determined for each strain before the test by spreading the appropriate dilutions of bacterial culture on antibiotic-containing LB agar plates and counting the colony forming units after 24 hours growth at 30°C.

### Preparation of standard chemicals

All heavy metal salts were at least of analytical grade (98%): mercury (II) chloride (HgCl_2_), methylmercury chloride (MeHgCl), cadmium (II) chloride (CdCl_2_), zinc (II) sulphate-7-hydrate (ZnSO_4 _× 7H_2_O) and copper (II) sulphate-5-hydrate (CuSO_4 _× 5H_2_O) were purchased from Riedel-de-Haën, lead (II) nitrate (Pb(NO_3_)_2_) was from Fluka and silver (I) nitrate (AgNO_3_) from J. T. Baker B.V. 1 M stock solutions of CdCl_2_, ZnSO_4 _× 7H_2_O and CuSO_4 _× 5H_2_O as well as 0.1 M of HgCl_2_, AgNO_3 _and Pb(NO_3_)_2 _were prepared in MilliQ water. 0.1 M stock solution of methylmercury chloride was prepared in DMSO. Before the induction measurements, serial dilutions of the stock solutions (geometrical factor 3.3) were done in MilliQ water.

### Measurements and calculations

Serial dilutions of heavy metal standard solutions in MilliQ water were pipetted onto white 96-well Cliniplate (Thermo Labsystems, Helsinki, Finland) (100 μl per well), and an equal volume of the bacterial suspension (preparation escribed above) was added and the plates were incubated for 2 h at 30°C (optimal for the induction of bioluminescence in sensor cells) without shaking. After incubation, luminescence was measured with plate luminometer Fluoroskan (Thermo Labsystems). All the measurements with sensor and respective control bacteria were performed as three independent experiments in duplicate. To determine the background luminescence (non-affected by added metals) for each sensor and control strain, MilliQ water was added to bacterial suspension instead of metal dilutions (6 replicates).

Response of either metal sensor or control bacteria was expressed as normalised luminescence (NL) calculated as in [63] according to the following formula:(1)

where L_B _is the background luminescence (luminescence of bacteria without added heavy metal) and L_S _the metal-affected luminescence of the respective bacterium. The NL of the control strains could be used to correct the luminescence of the sensor stains in slightly toxic, coloured or turbid samples.

The limit of determination in NL values (LOD_NL_) for the sensor bacteria was determined in every assay as follows:(2)

where  is the mean background luminescence value of the sensor (minimum six blanks included in each assay) and SD is the standard deviation. The LOD as mg of metal l^-1 ^was the metal concentration corespondng to LOD_NL _in the calibration curve.

In the case of the control strains, the inhibition of luminescence by metals was calculated by the following formula:(3)

The 2-h EC_50 _(concentration of metal causing 50% decrease in luminescence during 2 hours of exposure) was calculated from concentration-effect curves using log-normal regression.

## Authors' contributions

AI was active in planning of the study, carried out most of the molecular genetic studies and supervised the calibration of sensors. She also drafted the manuscript.

TR carried out transposonmutagenesis and calibration of sensors

AK participated in the planning of the work concerning general toxicity, in coordination and in revision of the manuscript.

## Supplementary Material

Additional file 1 **Strains and plasmids used in this study**. Relevant characteristics of the bacterial strains and plasmids used in this study are provided. Click here for file
