# Supplementary material for: A suite of recombinant luminescent bacterial strains for the quantification of bioavailable heavy metals and toxicity testing
Source: BMC Biotechnol. 2009 May 8;9:41. doi: 10.1186/1472-6750-9-41 (PMC2685376; doi:10.1186/1472-6750-9-41)
Supplement: Additional file 1 — Strains and plasmids used in this study. Relevant characteristics of the bacterial strains and plasmids used in this study are provided. [file 1472-6750-9-41-S1.doc]

# Additional file 1. Characterization of strains and plasmids used in this study

| Strain or plasmid | Relevant characteristics | Reference |
| --- | --- | --- |
| *Strains* |  |  |
| *Escherichia coli* MC1061 | *araD*139 Δ*(ara, leu)*7697 Δ*lacX*74 *galU* *galK* *hsdR*2 *strA* *mcrA* *mcrB*1 | [61] |
| *Escherichia coli* SY372λ pir | λ(*lac, pro*)*argE*(Am)*rif, nalA, revA56*(λpir) | [67] |
| *Escherichia coli* S17-1λ pir | *pro thi hsdR*514(R^+^M^-^)Δ*recA* RP4::2-Tc::Mu::KmTn7(Tp^R^ Str^R^) | [69] |
| *Staphylococcus aureus* RN4220 | *rsbU*-,*agr*- | [72] |
| *Bacillus subtilis* BR151 | *trpC*2 *lys*-3 *metB*10 | [73] |
| *Bacillus subtilis* BR151(pBL1) | BR151 bearing plasmid pBLI, which carries the gene for LacI repressor; Cm^r^ | [38] |
| *Pseudomonas fluorescens* OS8 | Rif^r^, isolated from soil polluted with toluates | [65] |
| *Plasmids* |  |  |
| pTetlux | *luxCDABE* (from *Photorhabdus luminescens*) under the control of P*tetA* | [62] |
| pmerR_BS_Bluc | pSL1190 (Amersham Pharmacia Biotech) backbone, *lucFF* under the control of P*mer; merR*_BS_ and *merB* (from pDU1358 in *Serratia marcescens,* [27]); Ap^r^ | [49] |
| p**merR_BS_BPmer**lux | same as pmerR_BS_Bluc, but *lucFF* replaced by *luxCDABE* (from *P. luminescens*) | This study |
| pSLcueRPcopAluc | pSL1190 (Amersham Pharmacia Biotech) backbone; *lucFF* under the control of P*copA ;cueR* (from *E. coli* chromosome, [28]); Ap^r^ | [63] |
| pSL**cueR** | P*copA* and *lucFF* removed from pSLcueRPcopAluc | [63] |
| pSLcueRPcopAlux | same as pSLcueRPcopAluc but *lucFF* replaced by *luxCDABE* (from *P. luminescens*) | This study |
| pDN**PcopA**lux | P*copA,* and *luxCDABE* from pSLcueRPcopAlux inserted to pDN18N [64], Tc^r^ | This study |
| pSLzntluc | pSL1190 (Amersham Pharmacia Biotech) backbone, *lucFF* under the control of P*zntA*; *zntR* (from *E. coli* chromosome, [29]); Ap^r^ | [50] |
| pSL**zntR** | P*zntA* and *lucFF* removed from pSLzntluc | This study |
| pSLzntRPzntAlux | same as pSLzntluc but *lucFF* replaced by *luxCDABE* (from *P. luminescens*) | This study |
| pDN**PzntA**lux | P*zntA* and *luxCDABE* from pSLzntRPzntAlux inserted to pDN18N [64], Tc^r^ | This study |
| pSLlux | pSL1190 backbone, *luxCDABE* (from *P. luminescens*) under *lac* promoter, Ap^r^ | [32] |
| pDNlux | pDN18N backbone, *luxCDABE* (from *P. luminescens*) under T7 promoter, Tc^r^ | [32] |
| pDN**merR_BS_BPmer**lux | *merR*_BS_, *merB*and P*mer* (from pDU1358 in *Serratia marcescens*), *luxCDABE* from p**merR_BS_BPmer**lux inserted to pDN18N [64], Tc^r^ | This study |
| pSLpbrRPpbrAlux | pSL1190 (Amersham Pharmacia Biotech) backbone, *luxCDABE* (from *P. luminescens*) under the control of P*pbrA*; *pbrR* (from *Ralstonia metallidurans* plasmid pMOL30 (GenBank CP000354), [33]); Ap^r^ | This study |
| pDN**pbrRPpbrA**lux | P*pbrA*, *pbrR and luxCDABE* from pSLpbrRPpbrAlux to pDN18N [64], Tc^r q^ | This study |
| pSLcadRPcadAlux | pSL1190 (Amersham Pharmacia Biotech) backbone, *luxCDABE* (from *P. luminescens*) under the control of P*cadA*; *cadR* (from *Pseudomonas putida* chromosome, [34]); Ap^r^ | This study |
| pDN**cadRPcadA**lux | P*cadA*, *cadR and luxCDABE* from pSLcadRPcadAlux to pDN18N [64], Tc^r^ | This study |
| pSB2025 | *luxABCDE* of *P. luminescens* with enhanced translational signals for Gram-positive bacteria | [37] |
| pTOO24 | shuttle vector, promoter of *cad*, *cadC* (from pI258), *lucFF* (from *Photinus pyralis*)*,* Kn^r^ | [36] |
| p**cadCPcadA**lux | *lucFF* in pTOO24 replaced by *luxCDABE* (from pSB2025) | This study |
| p602/22 | shuttle vector, T5 promoter-*lac* operator, Kn^r^, Cm^r^ | [71] |
| p602/22lux | *luxCDABE* (from pSB2025) inserted to p602/22 | This study |
| pTCR241Kn | based on suicide plasmid pTCR241 [68] with mini-Tn5 transposon system, Ap^r^; Kn^r^ | This study |
| pTCRKnlux | *luxCDABE* (from *P. luminescens*) under the control of *lac* promoter inserted to pTCR241Kn | This study |
| pTCRKnmerR_BS_BPmerlux | *luxCDABE* (from *P. luminescens*) under the control of P*mer*, *merR*_BS_ and *merB* (from pDU1358) inserted to pTCR241Kn | This study |
| pTCRKncueRPcopAlux | *luxCDABE* (from *P. luminescens*) under the control of P*copA*, *cueR* (from *E. coli* chromosome) inserted to pTCR241Kn | This study |
| pTCRKnzntRPzntAlux | *luxCDABE* (from *P. luminescens*) under the control of P*zntA*, *zntR* (from *E. coli* chromosome) inserted to pTCR241Kn | This study |
| pTCRKnpbrRPpbrAlux | *luxCDABE* (from *P. luminescens*) under the control of P*pbrA*, *pbrR* (from *Ralstonia metallidurans* megaplasmid pMOL30) inserted to pTCR241Kn | This study |
| pTCRKncadRPcadAlux | *luxCDABE* (from *P. luminescens*) under the control of P*cadA*, *cadR* (from *P. putida* chromosome) inserted to pTCR241Kn | This study |

Rif - rifampicin

Ap – ampicillin

Tc – tetracycline

Kn – kanamycin

Cm – chloramphenicol
